# Bisphosphonate- and disumab-related gingival disorders: case analysis from the U.S. Food and Drug Administration Adverse Event Reporting System

**DOI:** 10.3389/fendo.2024.1367607

**Published:** 2024-08-22

**Authors:** Lei Wang, Wei Zhang, Cheng-long Zhao, Zhong-hua Fu

**Affiliations:** Department of Pharmacy, Henan Provincial People’s Hospital, People’s Hospital of Zhengzhou University, School of Clinical Medicine, Henan University, Zhengzhou, Henan, China

**Keywords:** antiresorptive drugs, FAERS, gingival disorders, adverse drug reactions, bisphosphonates, denosumab

## Abstract

Prior research has indicated that bisphosphonates (BPs) can improve periodontal disease because of their anti-osteoporosis properties. *In vitro* studies have shown that BPs induce cytotoxicity, inhibit wound healing, and thus affect periodontal disease. Denosumab and BPs have alternative indications. BP and denosumab are not known to correlate with gingival disorders. We assessed such a relationship by applying Bayesian and nonproportional analyses to data in the US FDA Adverse Event Reporting System (FAERS) database. The study analyzed BPs and denosumab-reported incidents with preferred terms found in the narrow Standardized MedDRA Queries for gingival disorders. A total of 5863 reported cases of gingival disorders were associated with five BPs (alendronate, pamidronate, ibandronate, risedronate, and zoledronate) and denosumab. More than 15% of patients with gingival disorders related to BPs and denosumab other than denosumab were hospitalized over short- or long-term periods. Our findings indicated BPs and denosumab had significant reporting odds ratios (ROR), proportional reporting ratios (PRR), and information components (IC) with respect to gingival disorders. Pamidronate had the highest association (ROR = 64.58, PRR = 57.99, IC = 5.71), while the weakest association was found with denosumab (ROR = 3.61, PRR = 3.60, IC = 1.77). Significant associations were found between the six drugs and gingival pain, gingival recession, gingivitis, periodontal disease, and periodontitis. In conclusion, our comprehensive overview of the correlations, clinical characteristics, and prognoses of BPs and denosumab-related gingival disorders suggests that these issues deserve continued surveillance and appropriate management.

## 1 Introduction

Drugs that counteract bone resorption, known as antiresorptive drugs (ARDs), interfere with bone metabolism to reduce abnormal bone remodeling or bone resorption (1). The most commonly used ARDs are calcium supplements, bisphosphonates (BPs), hormones, inhibitors of receptor activator of nuclear factor-κB ligand (RANKL), and other categories. BPs exert their antiresorptive effects through the inhibition of osteoclasts, which helps to restrict bone resorption and preserve bone density (2), while RANKL inhibitors block the activity of a crucial mediator in the regulation of osteoclast activity (3).

BPs can be classified into two main groups: nitrogen-containing (N-BP) and non-nitrogen-containing (NN-BP), with the former being more potent and more commonly prescribed. A commonly used RANKL inhibitor is the monoclonal antibody denosumab. These drugs are widely used in the treatment of various skeletal disorders, such as osteoporosis, malignancy-associated bone disease, and Paget’s disease (4, 5). Denosumab offers convenient administration and lacks renal metabolism, making it a potential alternative to BPs for treating osteoporosis and tumor bone metastasis. The most common adverse events for both classes of drugs were hypocalcemia, pain, and flu-like symptoms (6).

Gingival disease can lead to infection of the periodontal tissues and progressive loss of alveolar bone, ultimately resulting in tooth loss in severe cases (7). While previous studies have demonstrated the potential efficacy of BPs in improving periodontitis (8–10), it is noteworthy that the use of denosumab and BPs is associated with rare osteonecrosis of the jaw (ONJ) (11), which can cause alveolar bone loss, tooth mobility, and exacerbation of gum disease symptoms. Systematic analyses of these potential connections have not yet been reported. Therefore, this pharmacovigilance study aims to perform such an analysis utilizing data from the US FDA Adverse Event Reporting System (FAERS).

## 2 Methods

### 2.1 Data source

Data were gathered from the US Food and Drug Administration Public Data Open Project (Open FDA), which sourced raw data from the FAERS database. Extraction of data was carried out with OpenVigil2.1, an open pharmacovigilance data extraction, mining and analysis tool specifically designed for use with the FAERS database (12). OpenVigil2.1 operates solely on cleaned FDA data, with most duplicates and reports containing incomplete information being deleted.

In FAERS, adverse events are coded using preferred terms (PTs) from the Medical Dictionary for Regulatory Activities (MedDRA) (version 25.0). A specific PT may be assigned to multiple high-level terms (HLTs), high-level group terms (HLGTs), and system organ classes (SOCs). In addition, all PTs representing symptoms, signs, investigations, or diagnoses with potential relevance can be categorized into meaningful groups using Standardized MedDRA Queries (SMQs) to define a medical condition of interest. The present study incorporated the narrow SMQ of gingival disorders, which comprised 26 PTs (**Table 1**).

**Table 1:** The PT term and code included in the narrow SMQ of gingival disorders.

The term of PT	Code	Scope
Dental root perforation	10085658	Narrow
Excessive gingival display	10084758	Narrow
Gingival atrophy	10018275	Narrow
Gingival bleeding	10018276	Narrow
Gingival blister	10049304	Narrow
Gingival discolouration	10018278	Narrow
Gingival discomfort	10077854	Narrow
Gingival disorder	10018280	Narrow
Gingival erosion	10018282	Narrow
Gingival erythema	10067419	Narrow
Gingival hyperpigmentation	10049580	Narrow
Gingival hypertrophy	10018284	Narrow
Gingival hypoplasia	10018285	Narrow
Gingival injury	10049300	Narrow
Gingival pain	10018286	Narrow
Gingival pruritus	10049306	Narrow
Gingival recession	10018290	Narrow
Gingival scar	10085097	Narrow
Gingival ulceration	10049398	Narrow
Gingivitis ulcerative	10018296	Narrow
Gingivitis	10018292	Narrow
Noninfective gingivitis	10074863	Narrow
Periodontal destruction	10034535	Narrow
Periodontal disease	10034536	Narrow
Periodontal inflammation	10072574	Narrow
Periodontitis	10034539	Narrow

### 2.2 Data extraction

This retrospective study gathered data from the FAERS database between Q4 2003 and Q2 2023. Nitrogen-containing BPs (alendronate, ibandronate, pamidronate, risedronate, and zoledronate) and a RANKL inhibitor (denosumab) were identified as primary suspects in the reports. Duplicate entries with the same Individual Safety Report number were eliminated prior to analysis. In this report, the SMQ-narrow and PT dimensions are used to analyze the association of gingival disease with BPs and denosumab.

### 2.3 Data mining

Following the fundamental principles of Bayesian and non-proportional analysis, we utilized reporting odds ratio (ROR) (13), proportional reporting ratio (PRR) (14), and Bayesian confidence propagation neural network (BCPNN) algorithms (15) to investigate the correlation between drugs and the negative impact of gingival disorders. The equations and criteria for the three algorithms are shown in **Table 2**; if any of the three algorithms met the criteria, it was considered a positive signal.

**Table 2:** Summary of major algorithms applied for signal detection.

Algorithms	Equation^#^	Criteria
ROR	ROR=(a/b)/(c/d) 95%CI=e^ln(ROR) ± 1.96(1/a+1/b+1/c+1/d)^0.5^	95% CI>1, N≥2
PRR	PRR=(a/(a+c))/(b/(b+d)) χ2 =∑[(O-E)2/E], [O=a, E = (a + b)(a + c)/(a + b + c + d)]	PRR≥2, χ2≥4, N≥3
BCPNN	IC=log_2_a(a+b+c+d)/((a+c)(a+b)) IC025=e^ln(IC)-1.96(1/a+1/b+1/c+1/d)^0.5^	IC025>0

^#^a: number of reports containing both the suspect drug and the suspect adverse drug reaction. b: number of reports containing the suspect adverse drug reaction with other medications (except the drug of interest). c: number of reports containing the suspect drug with other adverse drug reactions (except the event of interest). d: number of reports containing other medications and other adverse drug reactions.

ROR, reporting odds ratio; CI, confidence interval; N, the number of co-occurrences; PRR, proportional reporting ratio; χ2, chi-squared; BCPNN, Bayesian confidence propagation neural network; IC, information component; IC025, the lower limit of the 95% two-sided CI of the IC.

### 2.4 Statistical analysis

A descriptive analysis was carried out to summarize the clinical traits of patients suffering from gingival disorders linked to BPs and denosumab. The continuous variable was presented as mean ± standard deviation (SD), and the frequency variable was expressed as a percentage. Pearson’s chi-square or Fisher’s exact test were used to compare the intervention and hospitalization rates of various ARDs. All statistical analyses were performed using IBM^®^ SPSS^®^ Statistics (version 26). Results with a P value less than 0.05 were deemed statistically significant.

## 3 Results

### 3.1 Descriptive analysis

The clinical features of patients with BPs- and denosumab-induced gingival disorders are presented in **Table 3**. The median age of patients who received BPs was 63.2 ± 9.2 years, whereas the median age of those who received denosumab was 71.0 ± 10.7 years. Female patients were affected more often than male patients. Over 60% of patients using alendronate, ibandronate, pamidronate, risedronate, and denosumab came from the United States, compared with 42.7% for zoledronate. Reports associated with BPs were most highly concentrated from 2009 through 2013, and while reports associated with denosumab were concentrated from 2014 to 2018.

**Table 3:** The clinical characteristics of patients with BPs- and denosumab-related gingival disorders.

Characteristics	Reports (N, %)					
	alendronate	ibandronate	pamidronate	risedronate	zoledronate	denosumab
N	2811	152	508	49	1548	795
Age						
≤44years	107(3.8%)	1(0.7%)	25(4.9%)	1(2.0%)	34(2.2%)	6(0.8%)
45-59 years	1062(37.8%)	21(13.8%)	122(24.0%)	7(14.3%)	311(20.1%)	68(8.6%)
60-74 years	1119(39.8%)	32(21.1%)	129(25.4%)	17(34.7%)	423(27.3%)	221(27.8%)
≥75 years	302(10.7%)	29(19.1%)	31(6.1%)	13(26.5%)	204(13.2%)	197(24.8%)
unknown	221(7.9%)	69(45.4%)	201(39.6%)	11(22.4%)	576(37.2%)	303(38.1%)
Mean ± SD	61.7 ± 10.6	67.7 ± 10.5	60.0 ± 11.3	67.0 ± 12.7	63.8 ± 11.8	71.0 ± 10.7
Gender						
Female	2509(89.3%)	146(96.1%)	388(76.4%)	45(91.8%)	1018(65.8%)	665(83.6%)
Male	206(7.3%)	3(2.0%)	106(20.9%)	3(6.1%)	484(31.3%)	67(8.4%)
unknown	96(3.4%)	3(2.0%)	14(2.8%)	1(2.0%)	46(3.0%)	63(7.9%)
Reported countries						
United States	2637(93.8%)	111(73.0%)	375(73.8%)	33(67.3%)	661(42.7%)	495(62.3%)
Japan	16(0.6%)	6(3.9%)	78(15.4%)	7(14.3%)	327(21.1%)	71(8.9%)
Germany	26(0.9%)	11(7.2%)	6(1.2%)	0(0.0%)	75(4.8%)	34(4.3%)
Italy	6(0.2%)	1(0.7%)	4(0.8%)	3(6.1%)	63(4.1%)	11(1.4%)
France	6(0.2%)	9(5.9%)	5(1.0%)	1(2.0%)	48(3.1%)	17(2.1%)
Reported years						
2003-2008	487(17.3%)	29(19.1%)	38(7.5%)	8(16.3%)	609(39.3%)	0(0.0%)
2009-2013	2228(79.3%)	70(46.1%)	295(58.1%)	24(49.0%)	767(49.5%)	141(17.7%)
2014-2018	66(2.3%)	40(26.3%)	8(1.6%)	9(18.4%)	104(6.7%)	412(51.8%)
2019-2023	30(1.1%)	13(8.6%)	0(0.0%)	8(16.3%)	68(4.4%)	242(30.4%)
Route						
	Oral	Intravenous	Intravenous	Oral	Intravenous	subcutaneously
Indication/Comorbidity						
	osteoporosis(2165)	osteoporosis(70)	metastases to bone(178)	osteoporosis(27)	metastases to bone(570)	osteoporosis postmenopausal(206)
	osteopenia(725)	osteopenia(15)	breast cancer(101)	osteopenia(11)	multiple myeloma(230)	osteoporosis(154)
	osteoporosis prophylaxis(137)	osteoporosis postmenopausal(5)	multiple myeloma(76)	osteoporosis prophylaxis(5)	breast cancer(171)	metastases to bone(44)
	hypertension(105)	bone density abnormal(3)	breast cancer metastatic(34)	microscopic polyangiitis(2)	osteoporosis(135)	breast cancer(25)
	hypothyroidism(101)	bone disorder(3)	osteoporosis(33)	osteoporosis postmenopausal(2)	breast cancer metastatic(89)	prostate cancer(16)
Concomitant/Prior medicines						
	cholecalciferol(392)	omeprazole(9)	zoledronate(361)	alendronate (10)	dexamethasone(181)	calcium(60)
	levothyroxine(240)	calcium(9)	anastrozole(86)	levothyroxine(9)	pamidronate(168)	cholecalciferol(53)
	ibandronate(219)	alendronate(6)	capecitabine(85)	amlodipine(8)	docetaxel(140)	levothyroxine(23)
	risedronate(205)	levothyroxine(5)	paclitaxel(85)	calcium(8)	letrozole(115)	Magnesium carbonate(15)
	estrogens(149)	atenolol(4)	hydrocodone(74)	celecoxib(5)	capecitabine(83)	ergocalciferol(15)

The therapeutic uses of the drugs in this study were different. Alendronate, ibandronate, and risedronate were mainly used to treat osteoporosis and osteopenia and as prophylaxis for osteoporosis. Zoledronate, pamidronate, and denosumab were used to treat osteoporosis, but they were also used to treat primary tumors, including breast cancers, and metastases to bone. The most commonly reported concomitant and prior medications were calcium supplements, other BPs, levothyroxine, antitumor agents, and nonsteroidal anti-inflammatory drugs.

### 3.2 Bayesian and nonproportional analyses

From Q4 2003 through Q2 2023, a total of 13,165,131 cases were recorded, including 24,025 cases of gingival disorders. Signal detection of BPs and denosumab-related gingival disease is shown in **Table 4**. The numbers of reported cases of gingival disorders associated with alendronate, pamidronate, risedronate, zoledronate, ibandronate, and denosumab were 2811, 508, 49, 1548, 152, and 795, respectively. Based on the aforementioned criteria, all six drugs showed statistical significance in terms of ROR, PRR, and information component (IC). Of these, pamidronate had the strongest association with gingival disorders (ROR = 64.58, PRR = 57.99, IC = 5.71), while the weakest association was found with denosumab (ROR = 3.61, PRR = 3.60, IC = 1.77).

**Table 4:** Association of various BPs and denosumab with gingival disorders.

Drugs	N	ROR (95% two-sided CI)	PRR(χ2)	IC(IC025)
alendronate	2811	51.20(49.16,53.33)	47.36(112803.78)	5.34(5.28)
ibandronate	152	4.75(4.05,5.58)	4.72(440.13)	2.18(1.91)
pamidronate	508	64.58(58.86,70.86)	57.99(27846.37)	5.71(5.57)
risedronate	49	5.98(4.51,7.93)	5.93(195.69)	2.46(1.98)
zoledronate	1548	20.69(19.64,21.8)	20.02(26193.37)	4.19(4.10)
denosumab	795	3.61(3.37,3.88)	3.60(1441.09)	1.77(1.65)

**Figure 1:**
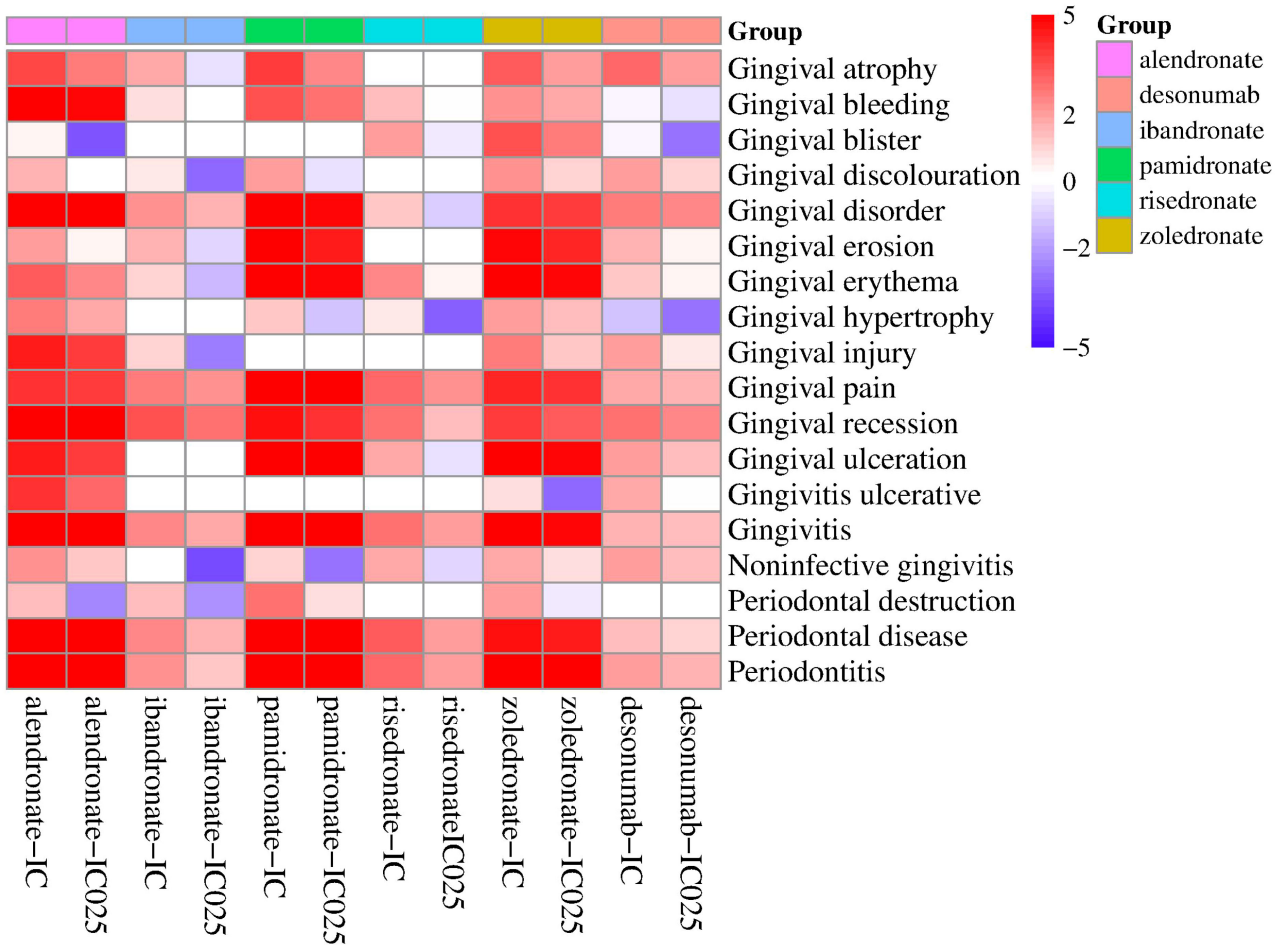
The IC values of BPs and denosumab in gingival disease.

The analysis focused on the reported occurrences of BPs and denosumab with PTs present in the narrow SMQ for gingival disorders, along with their corresponding PRR values (**Table 5**). Gingival bleeding, gingival disorder, gingival pain, gingival recession, gingivitis, periodontal disease, and periodontitis were the PTs that exhibited the most common occurrence in gingival diseases, accounting for more than 80% of the PTs. The PRR values of the drugs and the common PTs were greater than 2, and ibandronate, denosumab, and gingival bleeding were excluded. Adverse events with more than two reported cases were selected for correlation analysis with all drugs (**Figure 1**). The findings revealed high correlations between the six drugs and gingival pain, gingival recession, gingivitis, periodontal disease, and periodontitis.

**Table 5:** The correlation analysis of PTs included the narrow SMQ for BPs and denosumab with gingival disease.

adverse event	alendronate		ibandronate		pamidronate		risedronate		zoledronate		desonumab	
	N(%)	PRR(χ2)	N(%)	PRR(χ2)	N(%)	PRR(χ2)	N(%)	PRR(χ2)	N(%)	PRR(χ2)	N(%)	PRR(χ2)
Gingival atrophy	10(0.4%)	34.4(265.8)	2(1.3%)	13.2(11.8)	7(1.4%)	175.6(982.8)	/	/	8(0.5%)	21.9(129.7)	12(1.5%)	12.4(102.1)
Gingival bleeding	752(26.8%)	39.4(25338.7)	19(12.5%)	1.9(6.8)	35(6.9%)	12.5(356.4)	8(16.3%)	3.1(9.2)	121(7.8%)	4.7(345.6)	64(8.1%)	0.9(0.7)
Gingival blister	1(0.0%)	1.5(0)	/	/	/	/	2(4.1%)	24.6(24.5)	15(1.0%)	19.5(230.2)	2(0.3%)	0.9(0)
Gingival discolouration	4(0.1%)	4.7(8.1)	1(0.7%)	2.4(0)	2(0.4%)	17.4(16.6)	/	/	7(0.5%)	6.7(27.9)	13(1.6%)	4.6(31.7)
Gingival discomfort	3(0.1%)	3.6(3.3)	2(1.3%)	5(3)	/	/	/	/	/	/	4(0.5%)	1.4(0.2)
Gingival disorder	882(31.4%)	146.8(90432.7)	23(15.1%)	5.7(83.3)	65(12.8%)	58.9(3561.7)	3(6.1%)	2.9(2)	196(12.7%)	20.1(3310)	183(23.0%)	6.8(837.8)
Gingival erosion	4(0.1%)	7.9(17.3)	2(1.3%)	8.1(6.3)	22(4.3%)	362.3(6670.2)	/	/	32(2.1%)	60.8(1507)	7(0.9%)	4.1(13.1)
Gingival erythema	18(0.6%)	14.1(198)	2(1.3%)	3.1(1.2)	32(6.3%)	193.9(5549.7)	3(6.1%)	18.4(33.3)	84(5.4%)	62.4(4129.5)	12(1.5%)	2.7(11.1)
Gingival hypertrophy	26(0.9%)	7(124.3)	/	/	2(0.4%)	3.9(1.9)	1(2.0%)	2.1(0)	22(1.4%)	4.8(60.9)	5(0.6%)	0.4(4.2)
Gingival injury	21(0.7%)	60(993.8)	1(0.7%)	5.1(0.5)	/	/	/	/	6(0.4%)	12.5(50.1)	7(0.9%)	5.3(19.3)
Gingival pain	233(8.3%)	19.6(3883.3)	43(28.3%)	7.2(220.7)	114(22.4%)	70.1(7500.9)	17(34.7%)	11(144.9)	296(19.1%)	20.6(5126.5)	165(20.8%)	4(357.4)
Gingival recession	161(5.7%)	62.1(8199.4)	22(14.5%)	15.3(274.6)	24(4.7%)	60.6(1318.5)	6(12.2%)	16.1(70.1)	59(3.8%)	16.7(807.7)	82(10.3%)	8.6(500.3)
Gingival ulceration	41(1.5%)	33.5(1150.9)	/	/	33(6.5%)	198.6(5859.8)	2(4.1%)	12.2(10.8)	86(5.6%)	63.6(4296.2)	21(2.6%)	4.8(57.1)
Gingivitis ulcerative	13(0.5%)	50(505)	/	/	/	/	/	/	1(0.1%)	2.8(0.1)	5(0.6%)	5.2(12.4)
Gingivitis	762(27.1%)	77.2(47082.2)	33(21.7%)	5.7(123.8)	172(33.9%)	112.1(18084.1)	14(28.6%)	9.5(97.8)	529(34.2%)	40.918012.4	137(17.2%)	3.5(230.5)
Noninfective gingivitis	11(0.4%)	6.1(41.5)	1(0.7%)	1.1(0.2)	1(0.2%)	4.1(0.3)	2(4.1%)	8.9(7.2)	10(0.6%)	4.5(23.6)	27(3.4%)	4.5(67.9)
Periodontal destruction	1(0.0%)	17.9(3.3)	1(0.7%)	37.1(7.9)	3(0.6%)	447.4(794.6)	/	/	2(0.1%)	30.6(28.4)	/	/
Periodontal disease	731(26.0%)	334.5(125401.3)	14(9.2%)	6.9(64.6)	103(20.3%)	196.3(18466.1)	9(18.4%)	17.4(122.2)	157(10.1%)	33.7(4431.8)	41(5.2%)	2.9(48.8)
Periodontitis	615(21.9%)	190.5(75502.9)	13(8.6%)	5.5(43)	85(16.7%)	135.5(10677.3)	9(18.4%)	14.8(102.1)	306(19.8%)	60.8(14833.7)	74(9.3%)	4.6(195.5)
Periodontal inflammation	1(0.0%)	27.5(5.5)	/	/	/	/	/	/	1(0.1%)	22.4(4.3)	/	/
Gingival hyperpigmentation	/	/	/	/	1(0.2%)	134.2(31)	/	/	/	/	/	/
Gingival pruritus	/	/	/	/	/	/	/	/	1(0.1%)	8.5(1.2)	/	/

### 3.3 The rates of intervention and hospitalization linked to gingival disorders caused by BPs and denosumab

**Figure 2:**
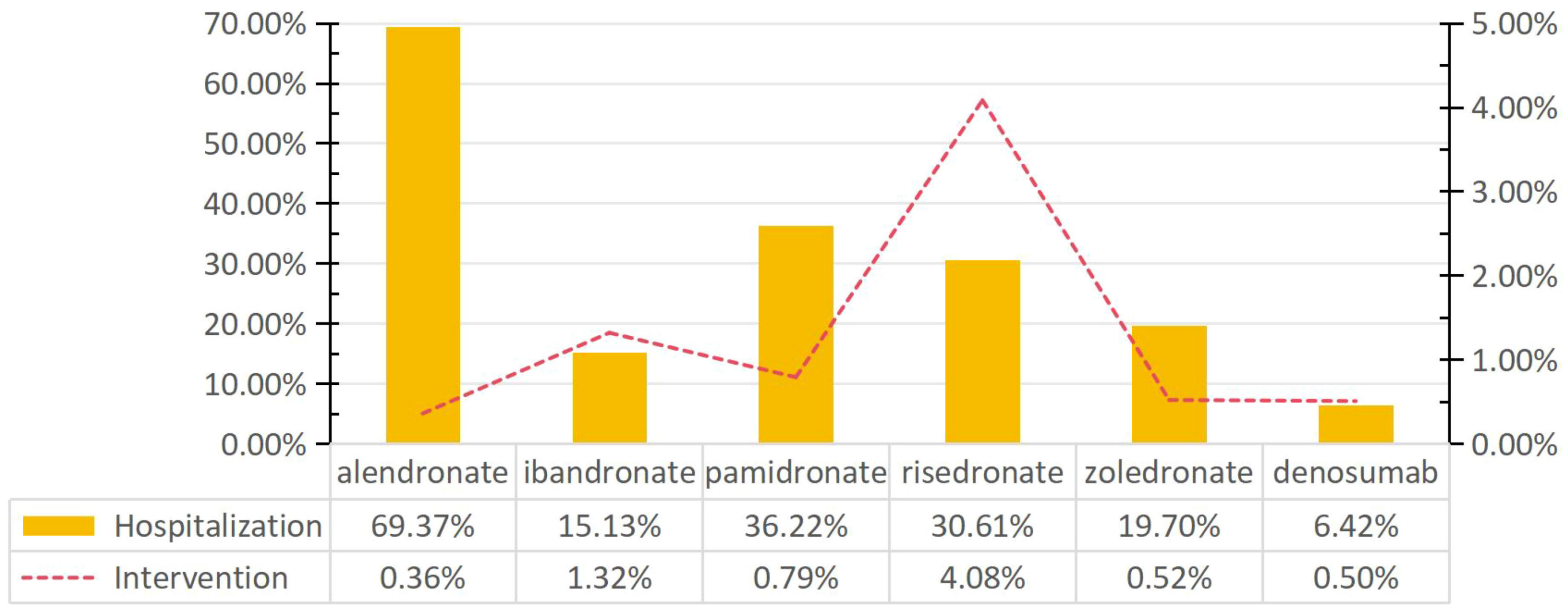
Rates of intervention and hospitalization for BPs- and denosumab-related gingival disorders.

To assess the impact of BPs and denosumab-related gingival disease, the proportion of patients who underwent intervention and/or were hospitalized was analyzed (**Figure 2**). Of the six drugs, alendronate was associated with the highest hospitalization rate for BP-related gingival disorders (69.37%). The rates associated with ibandronate, pamidronate, risedronate, zoledronate, and denosumab were 15.13%, 36.22%, 30.61%, 19.70%, and 6.24%, respectively. The intervention rate associated with risedronate was 4.08%, significantly higher than for other drugs.

## 4 Discussion

The purpose of this study was to analyze the association between BPs and denosumab with gingival disease, as well as the clinical characteristics and differences of the various drug interventions. To our knowledge, this analysis, which was based on FAERS database receipts from Q4 2003 to Q2 2023, is the first and largest such analysis of real-world data.

The study showed that BPs and denosumab were relevant to gingival disease. In the study of drugs, pamidronate and alendronate had strong correlations with gingival disorders, with respective ROR values of 64.58 and 51.20. In contrast, denosumab showed a relatively weaker association (ROR = 3.61). When analyzing the correlation of each drug with PTs included in the SMQ of gingival disorders, pamidronate and alendronate demonstrated the strongest correlations with the highest number of reported PTs (gingivitis, gingival pain, periodontal disease, periodontitis, gingival disorder, gingival bleeding, gingival ulceration, and gingival recession), while the lowest correlation was observed with denosumab.

Gingival lesions associated with BPs and denosamab are more prevalent in middle-aged and elderly women. The reported cases mainly fall within the 60-74 age group, and the proportion of alendronate-reported cases was 40%. A possible reason for these associations may be that the predominant indication for using these ARDs is post-menopausal osteoporosis. In addition, a reason why usage is lower in higher age ranges is that the metabolism of BPs depends on kidney function, and decreased renal function in older patients may increase the risk of adverse reactions. The management of osteoporosis and bone metastasis often requires prolonged use of ARDs. In clinical practice, other BPs are frequently administered sequentially for convenience or to mitigate adverse reactions. This study revealed that over 15% of patients who had used Alendronate, Pamidronate, and Zoledronate had previously been treated with other BPs, with Pamidronate accounting for 71.07%.

Gingivitis associated with BPs and denosamab often has a significant impact on quality of life, often requiring either short- or long-term hospitalization and other interventions to prevent permanent impairment or damage. More than 15% of patients with gingival disease associated with BPs required either short- or long-term hospitalization. In particular, alendronate had the highest hospitalization rate (69.37%), which was significantly higher than that of the other drugs (Pearson’s chi-squared test, P < 0.05). In contrast, the intervention rate for gingival disease related to alendronate was 0.36%, which was lower than that of other drugs. Furthermore, we observed that risedronate was associated with the highest risk of intervention for gingival disease compared to other drugs (4.08%, Pearson’s chi-squared test, P > 0.05).

Previous studies have indicated that BPs impede the healing process of the oral mucosa and lead to delayed wound closure (16). BP and denosamab may cause rare osteonecrosis of the jaw, which can lead to oral ulcers, alveolar bone loss, tooth loosening, etc. *In vitro* studies have provided additional evidence showcasing the genotoxic and cytotoxic effects of BPs on oral tissues (11). These agents have been shown to inhibit cell proliferation, metabolism, viability, and migration, alter cell morphology, and induce apoptosis and inflammation (17). The impacts of BPs on various types of oral cells, such as gingival fibroblasts, oral keratinocytes, periodontal ligament fibroblasts, periodontal ligament stem cells, and oral fibroblasts, have been investigated in several studies, and the findings suggest that BPs can lead to reduced cell proliferation, migration, and metabolism, as well as decreased viability and increased apoptosis (18–21).

N-BPs in particular have been found to inhibit the activity of transforming growth factor β (TGF-β) (23), which is an important factor in the differentiation of fibroblasts into myofibroblasts and thus the promotion of wound healing, cell migration, viability, and proliferation (22). Through this mechanism, N-BPs have been shown to impair re-epithelialization of oral mucosal tissue and to reduce wound healing (23). It has also been suggested that N-BPs can increase the production of reactive oxygen species (ROS) by inhibiting farnesyl pyrophosphate. ROS generation may regulate cell growth factors and signal pathways to reduce cell migration and proliferation (21). Furthermore, N-BP was found to down-regulate cyclin D1 expression in orofacial mesenchymal stem cells (24), resulting in cell division arrest at the G0/G1 phase and the hindering of cell proliferation. The induction of ROS generation by N-BP, as well as the upregulation of production of endogenous (TNF, TNF-receptor associated factor, and death domain) and exogenous (B-cell lymphoma 2, inhibitor of apoptosis, and caspase) apoptotic pathway-related factors in oral tissues (19), may explain the increased rate of gum tissue cell apoptosis (25).

Similarly, denosumab has been shown to inhibit the secretion of interferon-γ in gingival tissue and to enhance the cytotoxicity of natural killer cells (26), leading to an imbalance in the gingival microenvironment. Furthermore, denosumab upregulates the production of the pro-apoptotic factors Bad, Bax, and Bim, inhibits osteoclast activity, and delays the healing of oral bone injuries (27). The research investigating these mechanistic connections have mainly involved *in vitro* tests or experiments in non-human animals; therefore, additional clinical research or longer clinical studies are needed.

This study benefits from real-world research and data mining techniques; however, we also recognize several limitations. Firstly, our methods were unable to distinguish accurate data from false or inaccurately reported data. Secondly, while we were able to extract statistics from the basic patient information, the accuracy of concomitant diseases and medication histories was unclear, introducing potential confounding factors and uncertainties into our analysis. Thirdly, data mining using Bayesian and nonproportional analyses can only establish statistical associations and not causal relationships between adverse events and drugs.

Despite these limitations, our findings identified a significant association between reports of gingival disorders and patients taking BPs and denosamab. However, the lack of research on BPs and denosamab-related gingival disorders makes it challenging to establish a definitive link between the drugs and the adverse events. Further clinical, anatomical, or imaging studies involving large-scale human populations are necessary to provide more comprehensive explanations.

## Data Availability

The original contributions presented in the study are included in the article/supplementary material. Further inquiries can be directed to the corresponding author.
